# Fixation Stability as a Surrogate for Reading Abilities in Age-Related Macular Degeneration: A Perspective

**DOI:** 10.3390/jcm14227941

**Published:** 2025-11-09

**Authors:** Carolina Molin, Edoardo Midena, Enrica Convento, Giulia Midena, Elisabetta Pilotto

**Affiliations:** 1 Department of Neuroscience-Ophthalmology, University of Padova, 35128 Padova, Italy; carolina.molin@studenti.unipd.it (C.M.); edoardo.midena@unipd.it (E.M.); enrica.convento@unipd.it (E.C.); 2 IRCCS-Fondazione Bietti, 00198 Rome, Italy; giulia.midena@fondazionebietti.it

**Keywords:** age-related macular degeneration, retinal fixation stability, reading performance, reading speed, microperimetry

## Abstract

Age-related macular degeneration (AMD) significantly impacts central vision, fixation site and stability and reading abilities. This work aims to analyze the relationship between retinal fixation parameters measured using microperimetry and reading performance in patients with AMD. We identified the role of fixation stability measurement in the evaluation of reading abilities and discussed its implications both in clinical practice and in clinical trials. Our analysis highlights the importance of retinal fixation assessment as a precise surrogate for evaluating reading ability outcomes in AMD patients and as new clinical endpoint to demonstrate the functional effects of present and emerging target therapies.

## 1. Introduction

Age-related macular degeneration (AMD) is the most common cause of visual impairment related to central vision loss in developed countries [1]. The global incidence of AMD is increasing due to exponential population ageing worldwide [2]. Clinically, AMD is classified in the early and intermediate stages based on the presence of drusen and retinal pigmentary abnormalities, and in the late stage when macular neovascularization (MNV) and/or geographic atrophy (GA) develop [3]. In the early stage, which affects a wider portion of the AMD population, visual acuity and quality of life may not be significantly impaired. As the disease progresses to more advanced stages, the morphological macular changes compromise retinal sensitivity leading to the development of a central scotoma that impairs foveal fixation (both stability and site). At this point, patients experience difficulties in the performance of daily activities, particularly near ones, such as reading abilities, and consequently in vision-related quality of life [4]. To reduce visual function impairment, adaptive strategies occur, like moving fixation in an extrafoveal area, called preferred retinal locus (PRL) [5,6,7,8].

Microperimetry is currently the only objective visual function test that offers the possibility to quantify the sensitivity threshold of the macular region, as well as retinal fixation characteristics, including the location (central or eccentric) and stability, providing a direct correlation between anatomical and functional outcomes [9,10]. In particular, the area of an ellipse which encompasses fixation points for a given proportion of eye positions during a fixation test, known as the bivariate contour ellipse area (BCEA), has been shown to strongly correlate with reading performance, providing a more accurate assessment of fixation stability and allowing the detection of even minimal quantitative changes in the fixation area. A clinically stable fixation corresponds to a smaller BCEA, while for more unstable fixations, the BCEA will be larger [11,12]. For this reason, a complete understanding of retinal fixation stability changes and how its characteristics correlate to reading performance is essential in eyes with AMD, helping to identify patients who could benefit from earlier diagnosis, the detection of disease progression and treatment and/or rehabilitation options [13,14].

The aim of this perspective is to evaluate the relationship between reading performance and retinal fixation stability measurements obtained using microperimetry in AMD patients to provide recommendations on their application in both clinical and research settings.

## 2. Materials and Methods

The search for potentially relevant articles in the medical literature was conducted via MED-LINE from inception to August 2025 using the following search terms (queried both individually and in combination for advanced research): age-related macular degeneration, fixation stability, reading performance, reading speed and microperimetry. Only articles written in English were considered. Inclusion criteria were set to include journal articles and reports that provided relevant insights into the correlation between fixation stability measurements assessed using microperimetry and reading performance in AMD patients. An initial study in which fixation stability parameters were obtained using an eye-tracker was also included, due to its correlation with two subsequently analyzed studies. Exclusion criteria were non-original content and a lack of focus on retinal fixation stability with a correlation only between reading performance and PRL characteristics (location and distance from fovea). Additional articles were identified by reviewing the references of examined publications. Articles included in the reference list were fully examined by the authors.

## 3. Results

### 3.1. Reading Performance in AMD

#### 3.1.1. Reading Tests

The Minnesota Reading Acuity (MNREAD) charts and the RADNER reading charts are the most commonly tests adopted in AMD clinical trials, designed to determine reading performance (both acuity and speed) in one simultaneous examination. The MNREAD charts provide more sensitive and reliable measures of reading ability because their sentences are characterized by three lines with 60 characters including spaces and consist of ten standard-length words to minimize the variations in scoring that occur as result of the different word lengths found in different sentences. These charts are available in several languages and they provide the logMAR notation, the Snellen notation and M-units for 40 cm [15,16].

#### 3.1.2. Reading Parameters

The most useful parameters in the assessment of reading capacity in AMD patients are reading acuity (RA) and reading speed (RS). RA is defined as the smallest print size sentence that the patient can read without making significant errors. RS refers to the words per minute (wpm) that can be read across different print sizes until the patient does not complete reading the sentences [15]. According to some studies, an RS under 30 wpm is insufficient for sustained reading [14]. However, various methods to quantify RS have been described and three other reading parameters are commonly used: maximum reading speed (MRS), critical print size (CPS) and reading accessibility index (ACC). MRS is the fastest RS when not limited by the print size (larger that CPS). CPS is the smallest print size that can be read at the MRS, after which RS declines rapidly. ACC is the mean RS measured across the 10 largest print sizes [17]. Recently, additional RS measures have been proposed to better detect longitudinal visual function decline in patients with GA, such as the RS of the fastest sentence read (RS_1_) and the mean RS of the three fastest sentences read (RS_2_). It was demonstrated that ACC and RS_2_ were better than the two widely used RS measures (RS_1_ and MRS) in capturing progressive functional decline in eyes with GA [18].

#### 3.1.3. Reading Disabilities

Reading is a complex activity that requires the precise recognition of small details (letters and words), high-acuity vision, foveal integrity and a central and stable fixation [19,20]. However, reading ability is influenced by many other factors, unrelated to macular morphology and function, including oculomotor control, literacy level, cognitive factors, better or worse seeing eye, monocular versus binocular evaluation and the language’s directionality (left-to-right, right-to-left or top-to-bottom). In AMD patients, even when individual letters can be recognized, RS is often reduced due to slower scanning across lines of text. Decreased contrast sensitivity and visual acuity make small-print text particularly challenging, while the crowding effect (where surrounding letters interfere with word recognition) further increases reading errors [21]. Reading impairments become more pronounced with disease progression and are closely related to the stage of AMD [22]. In early and intermediate AMD stages, despite preserved central vision, subtle changes in macular sensitivity and contrast perception may induce mild reading disabilities, especially under suboptimal conditions (dim lighting and low contrast). In these situations, reading may become slower and difficult, although generally still possible without assistive tools [23,24]. As the disease progresses, associated with the significant disruption of foveal function, reading performance becomes significantly impaired, with differences between the atrophic and exudative forms. In patients with GA, reading abilities gradually decline as the atrophic area extends toward and involves the fovea, leading to central vision loss [25]. However, the slow nature of GA progression may allow patients to develop adaptive strategies, such as the use of magnification or eccentric vision, which can partially compensate for central vision loss and help to preserve residual reading ability. In contrast, exudative AMD often results in a more abrupt and severe decline in reading performance. Sudden central vision loss due to the pathological growth of new blood vessels, combined with hemorrhage, fluid accumulation and secondary fibrotic scarring in the macular area, can severely disrupt text scanning and word recognition [22]. With treatment which induces the reduction in the exudation, reading performance may improve. In a prospective case series of 30 eyes with exudative AMD, average RS improved from 59 wpm at baseline to 85 wpm after three intravitreal injections of anti-vascular endothelial growth factor (VEGF) [26]. However, even when treated with anti-VEGF therapy, many patients struggle to recover fluent reading, as the rapid onset of visual changes limits the development of compensatory mechanisms. As a result, patients with exudative AMD often experience greater reading difficulties compared with those with GA, with marked reductions in speed, accuracy and comprehension [22].

### 3.2. Fixation Stability in AMD

#### 3.2.1. Fixation Analysis

**Figure 1:**
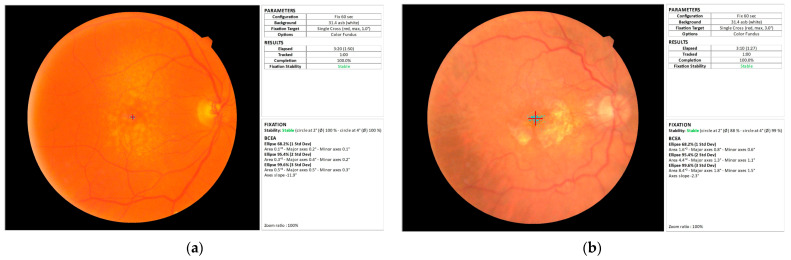
Fixation stability measurements automatically obtained by microperimetry in two patients with AMD. (**a**) Patient with intermediate AMD: BCEA is small and fixation is stable. (**b**) Patient with GA: BCEA is large and fixation is stable.

Fixation stability can be quantified using an eye-tracker or, more frequently, by microperimetry (also known as fundus-related perimetry) [27]. Fixation patterns (location and stability) are automatically recorded by microperimetry through two different methods: the clinical classification, a qualitative method proposed by Fujii et al. [28], and BCEA analysis, a quantitative method [29]. The clinical classification calculates the proportion of fixation points that fall within a certain distance from the centre of fixation. P1 and P2 values show the percentage of fixation points in a circle with a radius of 1° and 2°, respectively. According to this technique, fixation is defined as stable when more than 75% of the fixation points are located within a 2° diameter circle, centred on the mean fixation position of all points. Fixation is described as relatively unstable if less than 75% of the fixation points are located within a 2° circle but more than 75% fall within a 4° diameter circle, and as unstable if less than 75% of all fixation points are located within the 4° circle [28]. BCEA analysis is a more precise method that quantifies fixation stability by calculating the ellipsoidal area that includes a defined proportion of the fixation points: 68.2%, 95.4% or 99.6% of the points, which correspond to 1, 2 and 3 standard deviations (SD), respectively (Figure 1). Although both methods are useful in assessing fixation stability, BCEA analysis not only is more accurate than the clinical classification, but it can also detect even minimal quantitative changes in the fixation area [11,12,30]. Microperimetry can register fixation in two different modalities: as an isolated fixation task (static fixation) or during a full microperimetry sensitivity test (dynamic fixation) performed to assess the retinal sensitivity threshold. Dynamic fixation is influenced by its acquisition modality as the attention of the patient to the fixation target may be impaired or at least “confused”, contributing to more subtle signs of fixation instability, particularly in patients with a macular disease [11,31]. In a comparative study of 149 eyes with different macular diseases (60 with AMD) and 171 normal eyes, all pathologic groups showed more unstable fixation in the dynamic modality according to both the clinical and the BCEA methods (*p* < 0.0001). Instead, the change in fixation stability in the control group was highlighted in the dynamic modality only by BCEA analysis, which showed areas significantly larger than those obtained in the static fixation modality (*p* < 0.0001) [11].

#### 3.2.2. Fixation Impairments

Microperimetry provides critical insights into the fixation behaviour of AMD patients, highlighting distinct profiles between different forms of the disease. In particular, in patients with early and intermediate AMD, as also in healthy subjects, fixation is typically central (foveal) and stable [32]. Instead, the progression of AMD to more advanced stages, when central vision is lost due to the development of a dense and irreversible scotoma, can be functionally described by instability and the loss of central retinal fixation with the use of a PRL located outside the damaged foveal region (extrafoveal) [33]. In patients with GA, fixation tends to remain relatively central and stable in the earlier phases that leave the fovea unaffected (foveal sparing). As GA lesions gradually progress toward the fovea, patients often develop a PRL at the junction between the spared and atrophic retina [34,35]. It was observed that 77% of eyes with GA exhibited a PRL and this proportion increased to 91% after a follow-up period of 5.3 years, with 81% of eyes preserving the initial PRL location [36]. The slow progression of GA allows more time for adaptation and many patients exhibit eccentric but relatively stable fixation. In a longitudinal study of 20 eyes with GA, mean BCEA significantly decreased during a follow-up period of 12.3 ± 4.5 months, confirming that even if GA area progresses, fixation becomes more stable [37]. Microperimetry analysis in these patients frequently shows a moderate BCEA with a consistent PRL and training with biofeedback may further improve both fixation stability and reading performance [38]. On the other side, exudative AMD is associated with more unstable fixation, especially in the acute phase or in cases with persistent subretinal or intraretinal fluid. The sudden loss of foveal function due to exudation, hemorrhage or secondary scarring leads to the abrupt displacement of fixation. Patients often fail to develop a well-defined or functional PRL, resulting in a broader and more variable distribution of fixation points. Microperimetry analysis in eyes with exudative AMD typically reveals lower P1 percentages, larger BCEA and unstable fixation patterns [10]. These impairments may persist even after anti-VEGF treatment, because both central and stable fixation are demonstrated to be directly related to the absence of subretinal thickening or fibrosis, an intact subfoveal third hyperreflective band and an intact external limiting membrane [39].

### 3.3. Relationships Between Reading Performance and Fixation Stability in AMD

The effects of PRL location and distance from the PRL to fovea on reading abilities are still debated, with some authors reporting an association and others suggesting a lack of a significant relationship; however, reading performance data indicate that PRL characteristics have little impact on RS [40,41,42,43,44]. The anatomical location of the PRL, even if near the fovea, does not necessarily reflect visual function and can still provide reading impairments if fixation is unstable. On the other side, it has been widely demonstrated that fixation stability has a significant impact on reading performance, with both cross-sectional and longitudinal studies demonstrating a higher RS for patients with more stable fixation. Fixation stability provides a direct functional biomarker of the patient’s ability to maintain a stable retinal reference, which is essential during reading. Studies that have reported a relationship between fixation stability measurements obtained by an eye-tracker or microperimetry and reading parameters are reported in Table 1 and hereafter discussed.

Crossland et al. first investigated the relationship between RS and fixation stability in patients with newly developed macular disease at baseline and after 12 months of follow-up [45]. These authors studied only the better eye of 20 patients with AMD, 5 patients with juvenile macular disease (JMD) such as Stargardt or Best disease and 8 control subjects. RS was recorded using sentences with similar properties to the MNREAD cards and fixation stability was assessed for a period of 10 s in each of the five positions of gaze using an SMI gaze-tracker (SensoMotoric Instruments, Berlin, Germany). Then, BCEA was calculated encompassing 68% of all fixation points. They found a significant negative correlation between RS and BCEA for both control subjects (r = −0.66, *p* < 0.05) and patients with macular disease at the baseline (r = −0.45, *p* < 0.05) and at 12 months assessments (r = −0.51, *p* < 0.05). These findings indicated that poorer fixation stability is associated with a slower RS. One limitation of this study is that retinal fixation was assessed with an infrared eye-tracker, which has been shown to measure less stable fixation and thus a larger BCEA than those measured by a scanning laser ophthalmoscope (SLO). As the head is not restrained, with the chin and forehead in a rest position, some eye movements occur to compensate for the small head movements [46]. For this reason, a few years later, the same authors compared reading parameters to fixation stability values obtained with an MP-1 microperimeter (Nidek Technologies, Padova, Italy) in patients with late AMD [46]. They studied the better eyes of 25 patients with GA or MNV. Reading was assessed using five different measures: peak reading speed (PRS) and CPS with the MNREAD chart, RS and error rate with the European reading test (EUREAD) and RS with the Rapid Serial Visual Presentation (RSVP) of text. Fixation stability was quantified during a 30 s static fixation test in four different ways: the fixation quality score of the clinical classification (stable, relatively unstable or unstable), the percentage of fixations falling within a circle of 2° and 4° diameter and finally the BCEA was calculated encompassing 68% of fixation points. Only the stable fixation group had a significantly faster RS and a smaller CPS than those in the other two groups (relatively unstable and unstable), in which fixations were not related to any measures of reading performance. Moreover, the percentage of fixation points within a 2° and 4° diameter circle was not related to any of the reading parameters analyzed. Conversely, the fixation stability measured by BCEA was significantly correlated to RSVP RS (r^2^ = 0.3, *p* < 0.05), MNREAD PRS (r^2^ = 0.42, *p* < 0.01), MNREAD CPS (r^2^ = 0.24, *p* < 0.05) and EUREAD error rate (r^2^ = 0.39, *p* < 0.01).

Similar results were obtained in the study of Rubin et al., in which the relationship between reading parameters and fixation stability in patients with late AMD was investigated [47]. They studied the better seeing eyes of 34 patients with a diagnosis of bilateral AMD (24 with MNV and 10 with GA). The Reading Navigator test was used to determine RA and CPS, and RS was measured in characters per second (cps) using standardized paragraphs. These were composed of a series of longer text passages that, in the opinion of the authors, could be used to more accurately measure RS compared with the MNREAD and Radner tests, in which the passages are quite short. Fixation stability was quantified during a 30 s static fixation test using the local BCEA technique with a Rodenstock SLO-101 (Rodenstock, Dusseldorf, Germany). This technique uses a probability density map of fixation points to determine the number of PRLs and then calculate a BCEA for each locus. The local BCEA is the sum of these single BCEAs, as distinguished from the global BCEA which includes a given proportion of all fixation data. One of the main findings of this study was that the fixation stability calculated by local BCEA was significantly correlated with RS (r^2^ = 0.25, *p* < 0.005). An additional analysis demonstrated that patients with MNV read slower than those with GA (mean difference = 0.21 log cps, *p* < 0.05). However, there were no significant differences between groups for fixation stability, probably due to the small sample of patients. The correlation between local BCEA with the two other reading parameters (RA and CPS) was not analyzed. In the two newer studies, both Crossland et al. and Rubin et al., consistent with their previous work on newly acquired AMD patients, demonstrated a strong relationship between fixation stability measurements and reading parameters also in patients with late AMD, particularly by calculating BCEA [45,46,47].

Subsequently, other groups focused their studies on comparing functional reading measurements with both methods to quantify fixation stability. Calabrèse et al. investigated the relationship between MRS and fixation stability, stratified by the type of late AMD [48]. In particular, these authors studied 89 eyes (64 with GA and 25 with MNV) of 61 patients. Three MRS values were recorded for each eye using MNREAD-like French sentences. Fixation stability was quantified as the percentage of fixations within a 4° diameter circle during a static perimetry examination with an MP-1 microperimeter (Nidek Technologies, Padova, Italy). The main findings of the study were as follows: the MNV group had a higher MRS and more stable fixation than the GA group and MRS was significantly correlated with fixation stability in both groups (r = 0.27, *p* < 0.05). According to these authors, the cause of this difference between GA and MNV may be related to the different temporal progressions of the disease before leading to total central visual field loss, which probably induces different adaptation processes. Fixation strategies used by patients with GA when the central visual field is completely lost are strongly constrained by the past, when macular vision was partially functional. On the other hand, the development of fixation strategies in patients with exudative AMD is probably not influenced by any previous strategy and can therefore potentially evolve more flexibly. Amore et al. compared RS to fixation stability in a large cohort of 120 people with different causes of visual impairment, of which the largest group consisted of 47 patients with AMD at an unspecified stage [49]. RS was evaluated using MNREAD charts and fixation values of the better eye were obtained during a 30 s static fixation test using an MP-1 microperimeter (Nidek Technologies, Padova, Italy). In particular, fixation stability was quantified in three different ways: the clinical classification (stable, relatively unstable or unstable), the percentage of fixations falling within a circle of 2° and 4° diameter and finally the BCEA was calculated encompassing 68.2%, 95.4% and 99.6% of fixation points. They found that the poorest fixation stability and reading performance were in patients with AMD and that RS was significantly related to the clinical classification (r^2^ = 0.34, *p* < 0.05), the proportions of fixation points falling into a 2° (r^2^ = 0.51, *p* < 0.001) and a 4° diameter circle (r^2^ = 0.36, *p* < 0.001) and all three BCEAs (r^2^ = 0.39, *p* < 0.001). However, in a multiple regression analysis, the main findings of the study were not only that the most efficient way to predict reading performance was the most frequently used BCEA68 (r^2^ = 0.62, *p* < 0.01), but also that the size of the ellipse used was not important because RS was strongly negatively related to any size of BCEA (r^2^ = 0.61, *p* < 0.01 for BCEA95 and BCEA99). Giacomelli et al. focused their study on comparing functional reading measurements with the standard clinical classification method [50]. They investigated the association of reading abilities with fixation stability in the better-seeing eyes of 160 low-vision patients, 123 with AMD and 37 with diabetic macular edema (DME). Reading performance was measured with the Italian version of MNREAD charts to obtain RS and RA. Fixation stability was defined during a 30 s static fixation test as the percentage of fixation points that fall within a 2° diameter circle using an MP-1 microperimeter (Nidek Technologies, Padova, Italy). A limitation of this study was that BCEA methods were not used because it was designed and conducted before these measures were shown to produce a better correlation with RS than a 2° stability percentage. This could be the reason why they found that 2° fixation stability was only weakly related to RS (r = 0.23, *p* < 0.05) and RA (r = −0.24, *p* < 0.05). Altinbay et al. investigated fixation stability in the better-seeing eyes of 63 patients with late AMD [51,52]. Reading performance was assessed using the Turkish version of MNREAD charts to evaluate RA, CPS, MRS and ACC. Fixation stability was assessed during a routine microperimetry analysis with MAIA microperimetry (Centervue, Padova, Italy) and it was determined by the clinical classification method (stable, relatively unstable or unstable) and by the BCEA encompassing 63% (BCEA63) and 95% (BCEA95) of fixational eye movements. They found that the RA, CPS and ACC parameters were significantly correlated with MRS (*p* ≤ 0.001) and that there was a significant relationship between MRS and fixation stability obtained by the standard clinical classification (*p* = 0.003). However, a limitation of this study was that the correlation between reading parameters and fixation stability measured with BCEA63 and BCEA95 was not analyzed.

## 4. Conclusions

Fixation stability measures assessed using microperimetry have been demonstrated to be strongly related to reading parameters in AMD patients, particularly RS and CPS. However, variability in microperimetry protocols (static versus dynamic fixation test) and different fixation classifications (clinical classification versus BCEA analysis) make comparisons between studies sometimes difficult. Among the available approaches, the static fixation test and BCEA quantification appear to be the most useful microperimetry modalities and methods to evaluate reading performance in AMD patients, allowing the detection of even minimal quantitative changes in their fixation area. Reading parameters are demonstrated to be closely related to BCEA68, BCEA95 and BCEA99. The last two areas provide greater accuracy in predicting early fixation stability changes in patients with progressive AMD [53]. BCEA values are known to be influenced by different fixation assessment methods. For example, BCEAs obtained by SLO are smaller, meaning more stable fixations, than those derived from an infrared eye-tracker, as the participant’s head is stabilized by a chin rest. Extreme eye positions beyond ±3 SD of the fixation data, such as those occurring during blinks, can also affect BCEA measurements. Considering these findings, Nidek microperimeters (MP-1 and MP-3) could provide a more accurate quantification of BCEA than MAIA microperimetry, which includes all recorded data points (even far outliers) in the BCEA calculation. Considering inter-device variability, only the BCEA from the same type of microperimeter with the same fixation test duration should be used to monitor the progression of fixation stability in AMD patients [54]. Correlating fixation area with reading abilities may represent a meaningful functional endpoint to demonstrate the effects of emerging target therapies for AMD and may also open up new strategies for personalized disease management. The ability of microperimetry to quantify fixation stability may be a powerful tool to evaluate functional outcomes in AMD patients in both clinical and research settings. However, current microperimetry devices and testing modalities have limited its widespread application in AMD clinical evaluation. The improvement of fixation testing methodologies might improve the role of fixation analysis as a reliable visual function endpoint in the present and future management of patients with AMD.

## Figures and Tables

**Table 1:** Studies reporting relationship between fixation stability measurements and reading parameters in AMD patients.

Study	Design, No. of Patients	Macular Disease	Reading Tests	Instrument and Fixation Test	Reading Parameters	Fixation Methods	Significant Results
Crossland et al., 2004 [45]	Longitudinal, 25	New AMD and JMD	MNREAD-like sentences	SMI gaze-tracker	RS	BCEA68	→ correlation (***p* < 0.05**)
Crossland et al., 2009 [46]	Cross-sectional, 25	Late AMD	MNREAD charts, RSVP and EUREAD	MP-1, static test	RS, PRS, CPS and error rate	Clinical classification Fixations in 2° and 4° BCEA68	→ correlation with RSVP RS and CPS only for stable group (***p* < 0.05**) → no relation (*p* > 0.05) → correlation with CPS, RSVP RS (***p* < 0.05**), PRS and error rate (***p* < 0.01**)
Rubin et al., 2009 [47]	Cross-sectional, 34	Late AMD	Reading Navigator test	Rodenstock SLO-101, static test	RA, CPS and RS	Local BCEA	→ correlation with RS (***p* < 0.005**)
Calabrèse et al., 2011 [48]	Comparative, 61	Late AMD	MNREAD charts	MP-1, static test	MRS	Fixations in 4°	→ correlation (***p* < 0.05**)
Amore et al., 2013 [49]	Cross-sectional, 120 (47 with AMD)	AMD (stage not specified)	MNREAD charts	MP-1, static test	RS	Clinical classification Fixations in 2° and 4° BCEA68 BCEA95 BCEA99	→ correlation (***p* < 0.05**) → correlation (***p* < 0.001**) → correlation (***p* < 0.001**)
Giacomelli et al., 2013 [50]	Cross-sectional, 160 (123 with AMD)	Late AMD and DME	MNREAD charts	MP-1, static test	RA and RS	Fixations in 2°	→ correlation (***p* < 0.05**)
Altinbay et al., 2021–2022 [51,52]	Cross-sectional, 63	Late AMD	MNREAD charts	MAIA, dynamic test	RA, CPS, MRS and ACC	Clinical classification BCEA63 BCEA95	→ correlation with MRS (***p* = 0.003**) → not analyzed

Statistically significant *p*-value in bold. no.: number; ACC: reading accessibility index; AMD: age-related macular degeneration; BCEA: bivariate contour ellipse area; CPS: critical print size; DME: diabetic macular edema; EUREAD: European reading test; JMD: juvenile macular disease; MAIA: Macular Integrity Assessment; MNREAD: Minnesota Reading Acuity; MP-1: Micro Perimeter 1; MRS: maximum reading speed; PRS: peak reading speed; RA: reading acuity; RS: reading speed; RSVP: rapid serial visual presentation; SLO: scanning laser ophthalmoscope; SMI: SensoMotoric Instruments.

## Data Availability

The data presented in this study are available in the article. Eventual additional data are available on request from the corresponding author.

## Abbreviations

The following abbreviations are used in this manuscript:

ACC	Reading accessibility index
AMD	Age-related macular degeneration
BCEA	Bivariate contour ellipse area
CPS	Critical print size
cps	Characters per second
DME	Diabetic macular edema
EUREAD	European reading test
GA	Geographic atrophy
JMD	Juvenile macular disease
MAIA	Macular Integrity Assessment
MNREAD	Minnesota Reading Acuity
MNV	Macular neovascularization
MP-1	Micro Perimeter 1
MRS	Maximum reading speed
PRL	Preferred retinal locus
PRS	Peak reading speed
RA	Reading acuity
RS	Reading speed
RSVP	Rapid serial visual presentation
SLO	Scanning laser ophthalmoscope
SMI	SensoMotoric Instruments
VEGF	Vascular endothelial growth factor
wpm	Words per minute
